# Safety and Efficacy of Endoscopic Ultrasound-Guided Fine-Needle Aspiration Biopsy of Littoral Cell Angioma

**DOI:** 10.7759/cureus.61800

**Published:** 2024-06-06

**Authors:** Abdullah Aleem, Parvir Aujla, Blake Purtle, Nitish Mittal, Srinivas Ramireddy

**Affiliations:** 1 Department of Internal Medicine, University of Texas Health Science Center at Houston, Houston, USA; 2 Department of Gastroenterology and Hepatology, University of Texas Health Science Center at Houston, Houston, USA

**Keywords:** ct-guided biopsy, splenic lesion, splenic tumor, ultrasound-guided aspiration, endoscopic us-guided fine-needle aspiration and biopsy, endoscopic ultrasound (eus), fine-needle aspiration, spleen, biopsy, littoral cell angioma

## Abstract

Littoral cell angioma (LCA) is a rare vascular tumor of the spleen that often requires histopathological analysis for diagnosis due to non-specific imaging features. The current approach is either splenectomy or image-guided percutaneous biopsy which carries notable procedure-associated morbidity and limited accuracy. We present a novel case of LCA successfully diagnosed with endoscopic ultrasound fine-needle aspiration biopsy (EUS-FNAB), demonstrating its potential to reduce the morbidity associated with traditional percutaneous biopsy methods. This case highlights EUS-FNAB's advantage in minimizing complications and its effectiveness in diagnosing vascular tumors of the spleen, supporting its inclusion in the diagnostic algorithm for splenic lesions. Further cases are encouraged to explore EUS-FNAB's role in diagnosing rare vascular tumors such as LCA to establish its efficacy and safety profile.

## Introduction

Littoral cell angioma (LCA) is a rare vascular tumor that stems from the littoral cells that coat the spleen's red pulp sinuses. These cells are notable for their dual functionality: they are involved in both the creation of blood components and the immune response [1]. The typical immunohistochemical pattern of littoral cells has positive expression for CD-68, factor VIII, and CD-31 and negative for CD-34 [1-3]. Compared to other splenic tumors that originate from the splenic vasculature, such as hemangioendothelioma and angiosarcoma, LCA carries a low risk of malignant potential. Less than 150 cases of LCA have been documented to date with only three reported to have undergone malignant transformation [4]. The etiology is unknown; however, there have been reports of associations with co-existing malignancies and immunological disorders which warrants close monitoring once diagnosed [5].

LCA is often asymptomatic and incidentally discovered on cross-sectional imaging. If the tumor exerts mass effect or vascular compromise, patients can present with abdominal discomfort, fever, or malaise [1]. The imaging characteristics of LCA are non-specific as the tumor itself appears similar to other vascular tumors found in the spleen [6]. For a definitive diagnosis, a histopathologic exam and subsequent immunohistochemistry are required. Historically, splenectomy was the standard approach for diagnosis, primarily due to uncertainties regarding malignancy. Percutaneous biopsies of splenic lesions have been approached cautiously due to the inherent vascularity of the spleen, which increases the risk of bleeding. Percutaneous biopsy, guided by computed tomography (CT) or ultrasound (US), carries a notable complication rate, including hemoperitoneum, pneumothorax, and bleeding [7,8]. Although prior studies have demonstrated the safety of endoscopic ultrasound fine-needle aspiration biopsy (EUS-FNAB) for splenic lesions, there remains a dearth of evidence regarding its safety specifically for primary vascular splenic tumors, known for their elevated bleeding risk. Herein, we present a rare case of LCA diagnosed with EUS-FNAB, highlighting the potential of this minimally invasive technique for accurate diagnosis and sparing patients the morbidity associated with percutaneous biopsy methods.

## Case presentation

**Figure 1:**
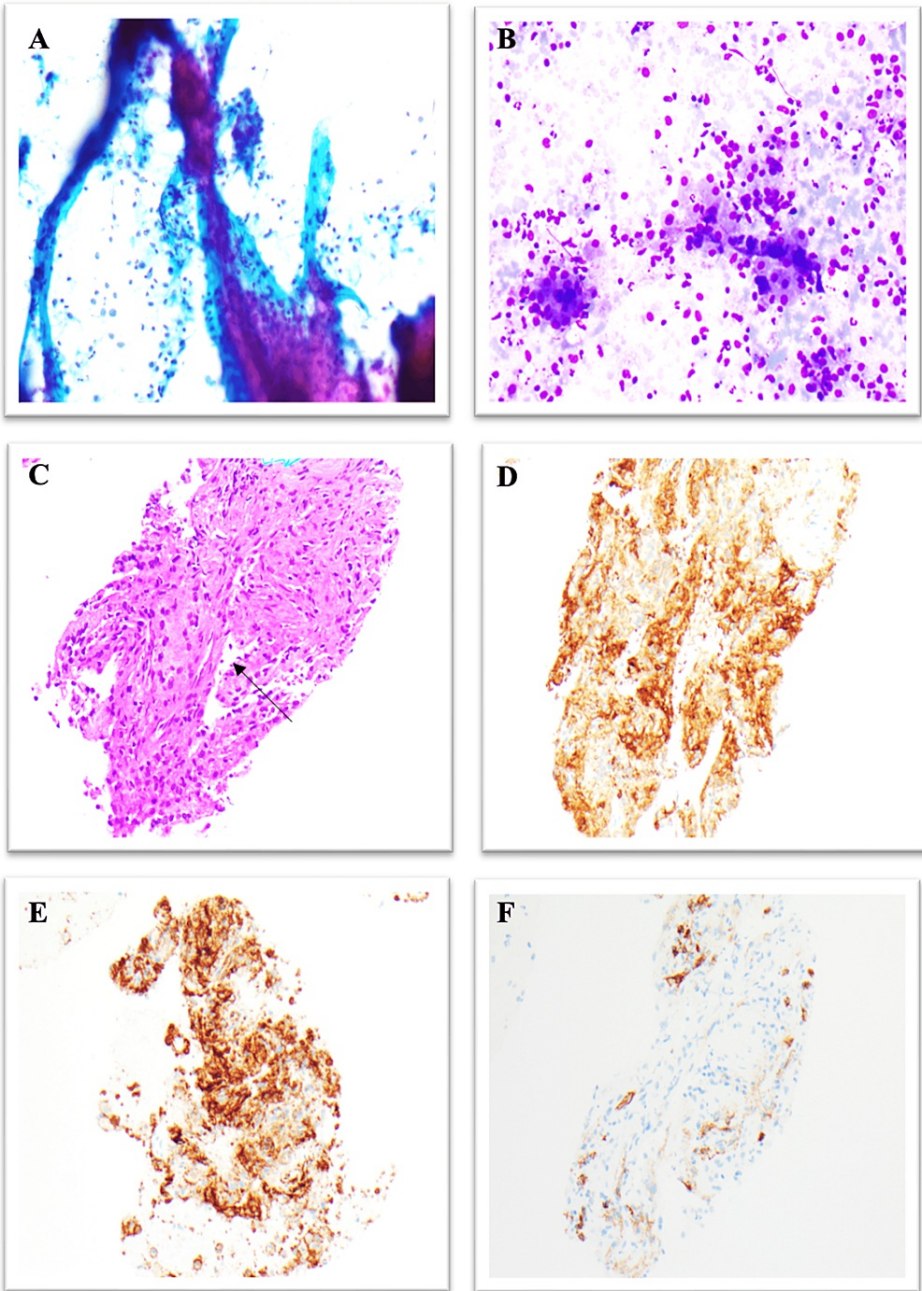
(A) Pap stain shows fibrovascular structures with spindle cells and plump cells with abundant vacuolated cytoplasm, magnification x 100. (B) Diff-Quik stain shows plump cells with abundant cytoplasm and background of scant spindle cells, magnification x 200. (C) H&E stain shows fibrovascular tissue with vascular channels lined by plump cells (arrow) without necrosis or significant cytologic atypia, magnification x 200. (D) CD4 immunostaining shows positive staining of the plump cells lining the vascular channels, magnification x 200. (E) CD31 immunostaining shows positive staining of the plump cells lining the vascular channels, magnification x 200. (F) CD34 immunostaining is negative in the plump cells lining the vascular channels, magnification x 200. Pap: Papanicolaou; H&E: Hematoxylin and eosin

A 42-year-old female with well-controlled systemic lupus erythematosus currently on hydroxychloroquine and belimumab was referred to a gastroenterology clinic for evaluation of gastroesophageal reflux disease and an incidental finding of a splenic mass seen on recent imaging. CT imaging showed a superior splenic subcapsular 8.3 cm x 7.3 cm mass along with multiple smaller than or equal to 1.5 cm splenic lesions. A small umbilical hernia and mild fatty diffuse infiltration of the liver were also noted. Subsequently, she went on to have a PET scan which did not show any hyperactivity around the splenic lesion. In the clinic, she complained of persistent reflux, unrelieved by omeprazole 40 mg daily. Additionally, she reported early satiety, shoulder pain, and night sweats. However, she denied weight loss, loss of appetite, or change in bowel habits. Physical exam was unrevealing, with no abdominal tenderness or organomegaly on palpation. The patient was recently seen by general surgery and was given the option of surgical splenectomy or an initial biopsy of the mass, she decided to proceed with biopsy to better determine etiology of splenic mass to deem if surgery was necessary. Endoscopic ultrasound showed a 6.2 cm x 7.1 cm hyperechoic poorly defined homogenous lesion within the spleen with surrounding normal splenic parenchyma. The patient subsequently underwent EUS-FNAB of the splenic mass using Doppler guidance to confirm avascular access, three passes of the mass were performed using a 22-gauge acquire needle. The aspirate smears showed fibrovascular tissue fragments with vascular structures lined by histiocytic cells, mixed with a background population of spindle cells, macrophages, and occasional megakaryocytes. Immunohistochemical stains of the cells lining the vascular channels were positive for CD-4, CD-31, CD-68, and factor 8 and negative for CD-34 consistent with a diagnosis of LCA (Figure 1). Although the dual vascular and histolytic differentiation was diagnostic of LCA, histological features and immunohistochemical analysis were essential in differentiating LCA from similar vascular tumors. Lack of malignant features such as increased mitotic activity and nuclear atypic ruled out littoral cell hemangioendothelioma. Both hemangiomas and LCA are positive for CD-31 however hemangioma is negative for CD-68. Hamartomas are frequently positive for CD-8, whereas LCA is CD-8 negative. Lymphangiomas often have cystic spaces lined by endothelial cells positive for VEGFR-3 which is absent in LCA. These distinct histopathological and immunohistochemical profiles facilitate accurate differentiation and diagnosis of LCA [2-4]. The patient did not have any post-procedural complications. Although malignant LCA is extremely rare, in this case, the patient eventually decided to undergo splenectomy to obviate any malignant potential.

 

 

 

## Discussion

Incidental findings of a splenic mass require biopsy with histological and immunohistochemical analysis to rule out tumors that may carry a high risk of malignancy. To our knowledge, this is only the second case of LCA successfully diagnosed using EUS-FNAB after Nagarajan’s case published in 2011 [7]. With the recent advancement of CT- and US-guided percutaneous biopsies of solid organs, most patients will opt for an initial biopsy prior to possible splenectomy for microscopic evaluation of a splenic mass. Efforts to reduce splenectomy for benign splenic lesions are needed due to the high morbidity rate of 8.6% to 37% post-splenectomy, mostly due to streptococcal infections [8]. Although percutaneous biopsies remain a viable option, they are not without their technical limitations and complication risks. Percutaneous US- and CT-guided biopsies of the spleen have been shown to have a complication rate between 5.2% and 8.2% [9-11]. The complications range from minor such as small volume hemoperitoneum and subcapsular hematoma to major such as colonic injury, renal injury, and pneumothorax. Technical difficulties are compounded by the presence of ascites, obesity, and prior abdominal surgeries [12]. Given the notable complication risk and technical challenges, it is prudent to consider alternative approaches when approaching splenic lesions. EUS-FNAB provides closer proximity to the spleen with trans gastric access while maintaining the ability to monitor needle movements in real time and the use of Doppler to avoid nearby vessels, making it a safe and effective modality.

In this case, a 22-gauge fine needle was used yielding high-quality tissue samples for histological and immunohistochemical analyses, which confirmed the diagnosis of LCA. CT- and US-guided percutaneous biopsies can be performed using either core or fine needles. Core needles, typically 18 gauge or larger, offer greater diagnostic accuracy, while fine needles are known for their superior safety profile, especially in patients with coagulopathies [7,11,13]. EUS-FNAB allows the provider to use a small caliber fine needle to minimize bleeding risk without compromising diagnostic accuracy due to excellent visualization of splenic lesions with EUS allowing for precise tissue sampling. Prior studies have shown that EUS-FNAB of splenic lesions is safe and accurate even for deep-seated and small lesions less than 25 mm [14]. A cohort of 15 patients successfully underwent EUS-FNAB of splenic lesions with 0 reported complications. The diagnoses in these patients were mostly tumors of lymphatic origin, there were no vascular tumors biopsied, which are expected to have a higher bleeding risk [12]. Our current case is pivotal as it contributes to the growing evidence supporting the safety of EUS-FNAB of vascular splenic tumors, underscoring the need for its increased availability to patients as a viable option.

## Conclusions

In conclusion, our case emphasizes the importance of EUS-FNAB as a safe and effective tool for diagnosing primary vascular splenic tumors such as LCA. By leveraging its technical advantages and diagnostic accuracy, EUS-FNAB holds promise as a cornerstone in the diagnostic algorithm for splenic lesions. Further research is needed to elucidate the role of EUS-FNAB in the diagnostic evaluation of rare vascular tumors such as LCA where bleeding is a major concern. Long-term studies assessing the diagnostic accuracy, complication rates, and clinical outcomes associated with EUS-FNAB will be instrumental in solidifying its role in the management of splenic pathology. For now, we encourage providers to consider EUS-FNAB when investigating splenic lesions, especially when patients are poor candidates for percutaneous biopsy methods.
